# Protocol for the Process Evaluation of a Complex, Statewide Intervention to Reduce Salt Intake in Victoria, Australia

**DOI:** 10.3390/nu10080998

**Published:** 2018-07-30

**Authors:** Kathy Trieu, Stephen Jan, Mark Woodward, Carley Grimes, Bruce Bolam, Caryl Nowson, Jenny Reimers, Chelsea Davidson, Jacqui Webster

**Affiliations:** 1 The George Institute for Global Health, University of New South Wales, Sydney, NSW 2042, Australia; sjan@georgeinstitute.org (S.J.); markw@georgeinstitute.org.au (M.W.); jwebster@georgeinstitute.org.au (J.W.); 2 Sydney School of Public Health, Faculty of Medicine, The University of Sydney, Sydney, NSW 2006, Australia; 3 The George Institute for Global Health, University of Oxford, Oxford OX1 3QX, UK; 4 Department of Epidemiology, Johns Hopkins University, Baltimore, MD 21218, USA; 5 Institute for Physical Activity and Nutrition, Deakin University, Geelong, VIC 3220, Australia; carley.grimes@deakin.edu.au (C.G.); caryl.nowson@deakin.edu.au (C.N.); 6 Department of Health and Human Services, Melbourne, VIC 3000, Australia; bruce.bolam@dhhs.vic.gov.au; 7 Victorian Health Promotion Foundation, Carlton, VIC 3053, Australia; jreimers@vichealth.vic.gov.au; 8 National Heart Foundation (Victorian Division), Melbourne, VIC 3000, Australia; chelsea.davidson@heartfoundation.org.au

**Keywords:** sodium reduction, public health nutrition, hypertension, process evaluation, disease prevention, population interventions

## Abstract

Systematic reviews of trials consistently demonstrate that reducing salt intake lowers blood pressure. However, there is limited evidence on how interventions function in the real world to achieve sustained population-wide salt reduction. Process evaluations are crucial for understanding how and why an intervention resulted in its observed effect in that setting, particularly for complex interventions. This project presents the detailed protocol for a process evaluation of a statewide strategy to lower salt intake in Victoria, Australia. We describe the pragmatic methods used to collect and analyse data on six process evaluation dimensions: reach, dose or adoption, fidelity, effectiveness, context and cost, informed by Linnan and Steckler’s framework and RE-AIM. Data collection methods include routinely collected administrative data; surveys of processed foods, the population, food industry and organizations; targeted campaign evaluation and semi-structured interviews. Quantitative and qualitative data will be triangulated to provide validation or context for one another. This process evaluation will contribute new knowledge about what components of the intervention are important to salt reduction strategies and how the interventions cause reduced salt intake, to inform the transferability of the program to other Australian states and territories. This protocol can be adapted for other population-based, complex, disease prevention interventions.

## 1. Introduction

Unhealthy diets are major causes of death and disability worldwide, second only to high blood pressure [1,2]. Improving dietary behaviours is challenging as the determinants extend beyond the individual-level and are influenced by social, economic and the environmental context. Dietary interventions are often complex, consisting of interconnected components, varying methods of delivery and multiple targets [3]. Therefore, it is often difficult to understand what components ensure success, how and why the interventions led to the observed effects and under what circumstances. Process evaluations can help explain this through examining the extent of implementation, causal mechanisms and context [4]. This can optimise future implementation of interventions. Despite the importance of process evaluations of complex interventions, they are not routinely reported.

Lowering population salt consumption is an example of an area that requires complex interventions. Excess salt consumption is a worldwide health problem, estimated to cause nearly 1 of every 5 premature CVD disease deaths [5]. Meta-analyses of trials consistently show reducing salt intake causes a dose-dependent reduction in blood pressure and thereby lowers cardiovascular risk [6]. Countries worldwide have been urged by the World Health Organization (WHO) to adopt feasible and effective interventions to reduce salt intake by 30% by 2025 for the prevention and control of noncommunicable diseases (NCDs) [7]. Recommended interventions include several interacting components targeting multiple influences that affect people’s eating behaviour: individual (knowledge, attitudes and behaviours), settings (work, school, communities, food outlets and supermarkets) and the macro-level environment (food supply, policies, economic systems, cultural norms) [8]. A systematic review identified that whilst there were 70 countries that implemented multi-component salt reduction strategies in 2014 [9], there were limited outcome evaluations of these interventions [10]. Of these, five demonstrated a significant mean decrease in salt intake from pre-intervention to post intervention, whilst the remainder showed no change or an increase [9,10]. The review concluded that there was uncertainty about what elements are most important to the success of strategies and that comprehensive evaluations, including process evaluations are needed to guide effective implementation in the future [9].

To date, little process evaluation research has been undertaken on population salt reduction interventions and the implementation procedures. A retrospective analysis of the successful UK salt reduction program aimed to highlight the key interventions components for other countries to follow [11]. More recently, a process evaluation of a salt reduction intervention in Fiji was conducted to understand why lower salt intake was not achieved and found that although the reach of campaign activities was high, most activities were one-off and there were no mechanisms for monitoring food industry’s adherence to the voluntary salt content targets once it was set up [12]. However, no protocols for process evaluations of complex salt reduction interventions have been published.

Population-wide salt reduction is crucial in Australia where average salt consumed is almost double the recommended amount of 5 g/day, causing increased blood pressure—the leading risk factor for death and disease in Australia [13]. This paper details the protocol for the process evaluation of a multi-component state-wide salt reduction intervention in Victoria, Australia. The process evaluation is integrated into a comprehensive evaluation of the effectiveness and cost-effectiveness of the Victorian Salt Reduction Partnership, a multi-faceted strategy that aims to reduce average salt intake of Victorians by 1 g/day. The intention of the comprehensive evaluation is to understand not only the question of ‘does it work?’ but also ‘what works?’, ‘how and why it works?’, ‘under what circumstances?’ and ‘at what cost?’. The role of the process evaluation is to understand how the interventions are functioning to enhance the implementation of the interventions in real-time but also to inform transferability and future implementation in other States and Territories in Australia, so that the WHO’s target of a 30% reduction in average salt intake can be achieved. To do this, the specific aims are:

1. explore the extent to which the interventions were implemented as planned;
2. examine how each intervention component contributed to overall goal of lower salt consumption relative to the causal pathways specified in the logic model and
3. identify and assess the relative influence of contextual factors that affect implementation and outcomes.

The aim of this study protocol is to describe the methodology of the process evaluation of the Victorian Salt Reduction Partnership, to promote transparency and share the approach, which can be adapted for use in other complex interventions.

## 2. Materials and Methods

### 2.1. Overview of the Outcome and Economic Evaluation

The process evaluation is nested in the broader study which is testing the impact and the cost-effectiveness of the Victorian Salt Reduction Partnership, a 4-year (2015–2019), multi-component strategy designed to lower average salt intake in Victorian adults and children by 1 g/day. The primary hypothesis is whether the Victorian Salt Reduction Partnership reduces average salt consumption between pre-intervention and post-intervention measures. The four secondary hypotheses are whether there is any change from pre-intervention to post-intervention in:

1. the knowledge, attitudes and behaviours of the Victorian population in regard to salt;
2. the reported use of discretionary salt during cooking or at the table;
3. the main food sources of salt in the diet and
4. the average salt content of processed foods.

In summary, the primary outcome will be evaluated through surveys of Victorian adults at baseline and again 3 years later (end of 2019). An age- and sex-stratified sample of 400 adults (aged 18–65 years) are recruited for each survey. Individuals previously enrolled in a Victorian study of salt intake in 2010 or 2014 [14] are contacted first, then additional participants randomly selected from the Victorian electoral commission, are recruited through a mailed invitation to achieve the full sample size. Twenty four hour (24 h) urine samples are collected for the primary analysis using standard procedures [14]. Dietary intake is also measured through a 24 h dietary recall in half of the sample, to estimate the main food sources of salt in the diet. Lastly, a structured questionnaire is administered to measure knowledge, attitudes and behaviours related to salt, discretionary salt use (during cooking or at the table) and exposure to the community-facing components of the Victorian Salt Reduction Partnership program. A previous study which measured 24 h urine samples, 24 h dietary recall and discretionary salt use habits in Victorian children between 2010 and 2013 will be used as the baseline measurement for children and will be repeated in mid-2018 to 2019 [15].

A cost-effectiveness analysis is also being undertaken to determine whether the intervention represents value for money compared to current practice (no specific salt reduction strategy), from a health sector perspective, with a view to informing the program’s transferability to other states and territories in Australia.

### 2.2. The Victorian Salt Reduction Partnership Intervention Strategy

**Figure 1:**
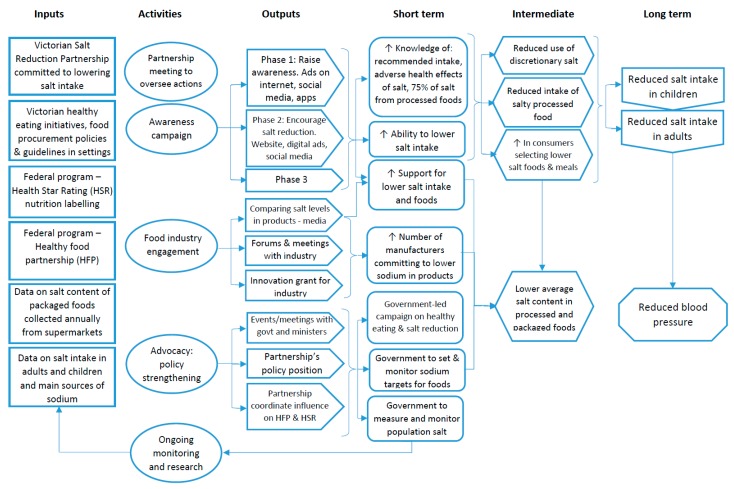
Logic model of the Victorian Salt Reduction Partnership program of interventions.

The Victorian Salt Reduction Partnership (referred to as The Partnership) is a multi-faceted strategy comprised of five key intervention components informed by previous state and community-level salt reduction interventions [16,17]. Ongoing research and evaluation are part of the Partnership’s strategy that informs and improves each of the interventions. The logic model, Figure 1, illustrates how each of the intervention components interact and the casual pathways to achieve reduced salt consumption in Victoria. The Victorian Health Promotion Foundation (VicHealth) and Heart Foundation are leading the implementation of the interventions (described below), with support from other collaborators of the Partnership.

#### 2.2.1. Strategic Partnership

The first component of the strategy was to develop a strategic partnership to increase the state-level coordination of salt reduction strategies and oversee the intervention actions. This involved building collaborations with non-governmental organizations (NGOs), health organizations, government and academia and identifying opportunities for coordinating and integrating salt reduction efforts into existing initiatives. A partnership of nine organisations formally provides strategic guidance for the intervention, with informal collaboration occurring with additional organisations.

#### 2.2.2. Increase Public Awareness to Improve Attitudes and Change Behaviours

The public awareness campaigns consist of several phases focusing on communicating messages about the health risk of high salt intake, current levels of consumption, sources of salt in the diet and approaches to lower salt intake, with an aim to improve attitudes to salt reduction action and the adoption of salt-lowering behaviours. The target audience is women aged 35–45 with children 0–12 years. The campaigns are informed by formative research about current knowledge, attitudes and behaviour related to salt in Victoria [18], previous behaviour change interventions and ongoing evaluations of each campaign phase among the target audience [19]. Static and digital ads, social media ads, a custom website and blogs are the primary paid channels of communication. Unpaid exposure for the campaign is generated via media coverage and organic social reach through the Heart Foundation.

#### 2.2.3. Policy Development and Strengthening

Another component of the Partnership’s strategy is to leverage existing federal and state-level healthy eating policies that relate to salt reduction and advocate for stronger government action. Using a situational analysis, existing policy initiatives were mapped, including nutrition standards for foods procured in public institutions, nutrition guidelines for catering, nutrition labelling on packaged foods (Health Star Rating Scheme) and the governments’ initiative to engage food industry to encourage healthy eating (Healthy Food Partnership) [20,21,22,23]. The Partnership will support these policy initiatives and strengthen the salt reduction component, so it is part of all existing healthy eating initiatives. In addition, the Partnership is advocating for the government to commit to three salt reduction actions:

1. establish sodium reformulation targets for foods;
2. establish surveillance systems to monitor sodium levels in foods and population salt intake; and
3. establish a national healthy eating campaign, including a focus on the importance of reducing salt intake.

#### 2.2.4. Innovative Approaches with Food Industry

In recognition that more than 75% of the average Australian’s salt intake comes from processed and packaged foods [24,25,26], the Partnership will make strategic investments and implement innovative approaches to engage both small to medium and large food manufacturers in lowering the salt content of food products. This includes educating food manufacturers to understand the need to reduce salt in packaged foods and demonstrating that the salt content can be reduced without loss of profits or market share and that there is a demand for such healthier foods. To influence manufacturers to produce lower salt products, the Partnership will hold meetings and forums with food industries, showcase manufacturers producing low salt options and communicate the variations in sodium content of products within food categories through media.

#### 2.2.5. Research, Monitoring and Evaluation

Formative and ongoing research is undertaken to inform and continually improve the design of the interventions. This includes research to understand Victorians’ knowledge and values related to salt to inform the key messages of the awareness campaigns, tracking and evaluation of the campaign to improve the next campaign phase, situational analyses to determine options to work with the food industry and monitor the food industries’ and organizations’ engagement with the Partnerships’ interventions.

### 2.3. Frameworks Informing This Process Evaluation

The process evaluation is conducted based on the UK Medical Research Council’s guidance on process evaluations of complex interventions [4]. This involves a comprehensive understanding of the implementation of interventions, how the context affects implementation and the outcomes, and the mechanisms of impact. Two theoretical frameworks developed for public health interventions and the translation of evidence into real-world settings were used to inform the process evaluation dimensions (Table 1). Guided by the framework proposed by Linnan and Steckler, the process evaluation examined reach, dose, fidelity and context [27]. This framework was chosen because it incorporates several frameworks, thereby ensuring its comprehensiveness and adaptability to complex interventions.

In addition, two dimensions were incorporated from the RE-AIM (Reach, Effectiveness, Adoption, Implementation and Maintenance) framework, which helps understand the impact of public health programs beyond the outcome or efficacy [28]. ‘Adoption’ which refers to the proportion and representativeness of organizations that adopt the intervention or policy, replaced ‘recruitment’ (procedures used to attract individuals or organizations to participate), as this is more relevant to the salt reduction interventions. ‘Effectiveness’ which refers to the positive and negative impacts of each intervention component was also included. Data on the cost of interventions collected for the cost-effectiveness evaluation, is also utilised in the process evaluation to demonstrate the level of implementation and information replicability.

### 2.4. Data Collection Methods and Analysis

A pragmatic mixed method approach to data collection will be adopted to minimise burden so as not to effect the delivery of the intervention whilst maximising understanding of the intervention processes. For each intervention component, specific indicators related to each evaluation dimension will be mapped (Table 2). Data collection methods are determined through firstly examining existing documentation and then identifying succinct methods to collect the remaining process data. The six main methods include:

1. routinely collected or administrative data;
2. surveys of Victorian adult population;
3. targeted campaign evaluation and tracking;
4. survey of the sodium content in packaged foods;
5. surveys of organizations, food industry and public institutions and
6. semi-structured interviews with intervention implementers, partnership members and key stakeholders.

Quantitative data collected from the surveys of the Victorian population and sodium levels in packaged foods will be analysed using the statistical program STATA IC version 13.0 for Window (StataCorp LP, College Station, TX, USA). Qualitative data will be uploaded and synthesised in matrices (as shown in Table 2) using NVivo 11. Quantitative data will be triangulated with the relevant qualitative data to inform the evaluation dimensions. For example, to understand the effectiveness of showcasing examples of reformulated food products, the quantitative measure would be whether there was a change in average sodium content in that targeted food category. This information will be considered in view of the interview responses to whether food industry stakeholders thought the case studies were useful in motivating them to lower the salt content of their products.

#### 2.4.1. Routinely Collected or Administrative Data

Routinely collected data consist of information provided regularly by the intervention implementers such as the partnership and working group meeting minutes, action plan and status update reports, media reports and costing information. Action plan reports, meeting minutes or status updates are used to collect detailed data on what interventions were delivered. Reports detailing the estimated audience reached through mass media, website clicks, social media activity and any public dialogue (for example government Hansards) supporting salt reduction are collected after each media release, campaign phase or advocacy event. In conjunction, Excel templates were purposely designed to collect detailed information on the activities implemented, personnel involved in delivery of interventions, time taken and the corresponding costs from a health sector perspective. Each organization will complete a separate template about the intervention component they are involved in implementing. Routinely collected data aims to inform *reach, dose delivered, fidelity, effectiveness and costs* of all the intervention components. These documents will be uploaded and synthesised in NVivo.

#### 2.4.2. Surveys of the Victorian Adult Population

Structured questionnaires administered pre-intervention, during the intervention and post-intervention will be used to collect data on exposure to the community-facing components of the intervention and the population’s knowledge, attitudes and behaviours (KAB) related to salt. Parents/caregivers for children less than 18 years are asked an additional seven questions about their attitudes and behaviours related to salt intake in children. A goal of approximately 2000 individuals, aged 18–65 years old, that are representative of the Victorian population in terms of age, gender, socio-economic status, and locality (metropolitan vs remote areas) will be recruited. In 2015 (pre-intervention), recruitment methods involved intercept surveys in shopping centres, recruitment through social media and a consumer research panel. Full details about the cross-sectional survey have been previously published [18,29]. The repeated mid-point surveys conducted in early 2018 and 2019 will aim to recruit comparable samples of 2000 individuals using the consumer research panel method only, as it was the most reliable method for recruiting participants from a range of demographic groups in 2015. The end-point survey conducted in 2020 will utilise the consumer research panel method and the intercept surveys in shopping centres. The surveys provide insight into the *reach, dose received* and *effectiveness* (change in knowledge and self-reported salt-lowering behaviours) of the community-facing intervention components. Multivariable regression analyses will be used to assess if there are differences in reach, dose and effectiveness between different demographic and clinical characteristics including age, gender, rurality, ethnicity, education status, household responsibility for grocery shopping, history of cardiovascular disease and use of antihypertensive medication.

#### 2.4.3. Targeted Campaign Tracking and Evaluation

In addition to the surveys of the Victorian adult population, an independent consumer research group are being commissioned to evaluate the *reach, dose, effectiveness*, *fidelity and contextual influences* of each campaign phase and their key messages amongst the target audience (parents aged 35–45 with children 0–12 years). A series of evaluations will take place after each campaign phase. This information is used to improve the next campaign phase and ensure the key messages have been understood before progressing to the next phase of behaviour change messages. These evaluations are also used as short-term outputs that are reported to the VicHealth Executive Board.

#### 2.4.4. Surveys to Food Industry Organizations and Public Institutions

Brief structured questionnaires will be sent out to food-industry related organizations (food manufacturers, importers, caterers, retailers) and public institutions (schools, hospitals, government agencies) with a goal to understand their exposure and engagement with the Victorian Salt Reduction Partnership program, their knowledge and actions relating to sodium levels in foods. The surveys will aim to include a representative sample or a wide range of different types of food industry organizations. Data from these questionnaires together with routinely collected data on food industry organizations and public institutions will inform *reach* and *adoption*.

#### 2.4.5. Sodium Content in Packaged Foods

Surveys of processed and packaged foods and their nutritional information will be systematically collected from the four major supermarkets in Australia each year. Data from each product will be collected and entered into a database using FoodSwitch [30]. The sodium content in foods will be analysed by food category and food company/manufacturer each year. This information, in conjunction with the questionnaire responses from food companies and routinely collected data about which food companies have been engaged or have committed to lowering the sodium content in their products, will generate information about the *effectiveness* of the food industry engagement intervention.

#### 2.4.6. Semi-Structured Interviews

Semi-structured interviews will be conducted with members of the strategic partnership, state and federal government, the food industry and NGOs working in health during the intervention and again post-intervention. Interviews with food industry organizations will aim to cover a representative sample of organization types. The semi-structured interviews seek to understand the contextual influences (facilitators and barriers) that may affect implementation and the outcomes for each intervention component. The contextual factors may be political, social, environmental or economical. The semi-structured interview responses will provide information on *context, fidelity* and *adoption*. Semi-structured interview responses will be transcribed and a thematic analysis will be undertaken based on each intervention component and the relevant evaluation dimensions in NVivo. The qualitative data will be used to provide context for the quantitative results.

## 3. Discussion

This study protocol presents the approach and methods used to comprehensively evaluate the process and extent of implementation of a complex, state-wide salt reduction strategy to assist with the interpretation of the project outcome. By assessing several dimensions of the implementation of interventions, we will help build an understanding about the degree of the implementation required to produce the individual intervention effects. This will contribute to new evidence on the potential effects of each individual intervention component (strategic partnership, campaign, industry engagement, advocacy and research and monitoring) and how they function within the context, to generate the observed changes in population salt intake. This will advance our knowledge about the key elements of a successful salt reduction intervention and its transferability to other states and territories in Australia. This protocol also provides a detailed example of how to embed a process evaluation within a comprehensive evaluation of a complex, public health nutrition intervention which are rarely reported but are urgently needed for successful implementation [31].

### 3.1. Dissemination

The findings of the process evaluation are delivered back to the Victorian Salt Reduction Partnership implementation team from the first year of implementation with updates every 3 to 6 months, so adaptions to the intervention can be made. Data from the process and the overall evaluation are disseminated through various approaches including media, community audiences, meetings with policy makers, publications and conference presentations to national and international audiences. In addition, the process evaluation findings will be disseminated to other state and territory-level governments to encourage adoption or support for similar multi-component salt reduction interventions.

### 3.2. Ethics

Ethical approval for the outcome and process evaluation was obtained from Deakin University Human Ethics Advisory Group (Project No.: HEAG-H 83_2015 & Project No.: HEAG-H 01_2019), the Human Research Ethics Committee at the University of Sydney (2016/770) and the Victorian State Government Department of Education and Training (Project No.: 2018_003666). All survey and interview participants are provided with a participant information sheet and are required to provide written consent.

### 3.3. Project Status

The Victorian Salt Reduction Partnership launched in May 2015. The first baseline survey of Victorian adults knowledge, attitudes and behaviours related to salt was conducted during September to November 2015 [18]. A National Health and Medical Research Council Partnership grant was awarded in 2016 to undertake a comprehensive evaluation of the program. The baseline assessment of 24 h urinary sodium excretion and knowledge, attitudes, 24 h dietary intakes and behaviours related to salt in Victorian adults was undertaken from around December 2016 to February 2017. Baseline information on 24 h urinary sodium excretion among children was conducted in 2010–2013 [15]. The process evaluation started in 2016 since the first phase of the public awareness campaign was implemented in May 2016 following formative research. This includes routinely collected administrative data, targeted campaign evaluation and sodium content in packaged foods. The first set of semi-structured interviews with stakeholders were conducted in March to May 2017. The mid-point survey to collect data on Victorian adults’ exposure to the campaign and the knowledge, attitudes, behaviour survey was completed in April 2018 and scheduled to be repeated in April 2019 and 2020. The end-point survey of Victorian adults’ salt intake is scheduled for December 2019 to February 2020 and July 2018 to April 2019 for the survey of Victorian children.

## Figures and Tables

**Table 1:** Summary of process evaluation dimensions assessed and the data sources.

Process Evaluation Dimensions	Definitions	Data Sources
Reach	The number or proportion of the intended target audience that comes into contact with the intervention [27]	Routinely collected data Population survey Organization survey Campaign evaluation
Dose (delivered or received)	The quantity or amount of each intervention component delivered or provided and to what extent did participants actively engage with the intervention [27]	Routinely collected data Population survey Organization survey Campaign evaluation
Fidelity	The extent to which the intervention was delivered as planned in relation to quality and integrity of the intervention as conceived by the developers [27]	Campaign evaluation Routinely collected data Population survey Qualitative
Effectiveness	The positive and negative impacts of the intervention component (in addition to the main outcome measures of the study) [28]	Routinely collected data Population survey Organization survey Campaign evaluation Salt content of foods Qualitative
Adoption	The proportion and representativeness of organizations that adopt the intervention or policy [28]	Routinely collected data Qualitative Organization survey
Context	Factors external to the intervention (social, political or economic environment) which may influence intervention implementation or outcomes [27]	Qualitative Campaign evaluation Population survey
Cost	The cost of each intervention component from a health sector perspective	Routinely collected data Qualitative

**Table 2:** Process evaluation dimensions, research questions and data collection methods.

Intervention Component	Reach	Dose/Adoption	Fidelity	Effectiveness	Context
Strategic Partnership	How many organizations are involved in the partnership? ^RD^	How well are the organizations engaging with the Partnership? ^RD, Qual^	Did the strategic partnership undertake its intended role? ^RD, Qual^	What are the stakeholders’ thoughts about the strategic partnership and whether it improved coordination and increased the number of salt reduction activities? ^Qual^	What were the contextual facilitators or barriers affecting the strategic Partnership? ^Qual^
Public awareness campaigns	What number or proportion of the target audience have been exposed to the campaigns? What subgroups did the campaign have higher or lower reach in? ^PopQ, CE^	What was the average number of sources of exposure to the campaign? What subgroups have higher or lower dose of exposure to the campaign? ^PopQ, CE^	How well did the target audience engage with the campaign? What proportion of the audience recall the key messages? Does the audience believe the campaign messages? ^CE^	What proportion of the target audience who were exposed to the campaigns, reported adopting the recommended salt-lowering behaviours? Were there changes in knowledge, attitudes and behaviours related to salt following the campaigns compared to baseline? Did the campaign generate consumer demand for lower salt foods? What subgroups were the campaign more or less effective for? ^PopQ, CE^	What were the facilitators or barriers affecting the implementation of the awareness campaigns? What were the contextual factors (social, environmental, economical) influencing consumers’ eating behaviors? ^Qual, CE, PopQ^
Innovative approaches with food industry	How many food companies have been exposed to the intervention? ^RD, OrgQ^	How many food companies have committed to lowering the sodium content of their food products? ^RD, OrgQ^	Were the interventions to engage food industry delivered as planned? ^RD, Qual^	Were there changes in the sodium content of food products of manufacturers that were targeted in the intervention? Were there changes in sodium content of food categories that were targeted by the intervention? Of the food companies that were engaged, were there changes in the sodium content of their products? Did the industry interventions influence food industry to lower the salt content of their foods? ^SL, RD, Qual^	What were the facilitators or barriers affecting the implementation of the activities with the food industry? What were the contextual factors influenced the sodium content of food products? ^Qual^
Policy development and strengthening (Advocacy)	How many Victorian or Federal MPs or government members have attended or were reached through the advocacy activities? ^RD^	How many advocacy events or meetings were held with policy makers? ^RD^	Were the advocacy interventions delivered as planned? ^RD, Qual^	How many government officials have publicly supported salt reduction? Is there evidence that any of the 3 policy asks will be adopted? Is there evidence of salt reduction being integrated into existing government healthy eating policies or initiatives? ^RD, Qual, OrgQ^	What were the facilitators or barriers affecting the implementation of the advocacy initiatives? What contextual factors (e.g., political) affect the adoption of the 3 policy asks? ^Qual^

RD—routinely collected data; PopQ—survey of the Victorian adult population; CE—campaign evaluation; SL—salt levels in packaged foods; OrgQ—organization questionnaire; Qual—semi structured interviews.
